# Effects of Food Availability on Yolk Androgen Deposition in the Black-Legged Kittiwake (*Rissa tridactyla*), a Seabird with Facultative Brood Reduction

**DOI:** 10.1371/journal.pone.0062949

**Published:** 2013-05-13

**Authors:** Z M. Benowitz-Fredericks, Alexander S. Kitaysky, Jorg Welcker, Scott A. Hatch

**Affiliations:** 1 Biology Department, Bucknell University, Lewisburg, Pennsylvania, United States of America; 2 Department of Biology and Wildlife, Institute of Arctic Biology, University of Alaska Fairbanks, Fairbanks, Alaska, United States of America; 3 Norwegian Polar Institute, Tromsø, Norway; 4 Alaska Science Center, United States Geological Survey, Anchorage, Alaska, United States of America; University of New England, Australia

## Abstract

In birds with facultative brood reduction, survival of the junior chick is thought to be regulated primarily by food availability. In black-legged kittiwakes (*Rissa tridactyla*) where parents and chicks are provided with unlimited access to supplemental food during the breeding season, brood reduction still occurs and varies interannually. Survival of the junior chick is therefore affected by factors in addition to the amount of food directly available to them. Maternally deposited yolk androgens affect competitive dynamics within a brood, and may be one of the mechanisms by which mothers mediate brood reduction in response to a suite of environmental and physiological cues. The goal of this study was to determine whether food supplementation during the pre-lay period affected patterns of yolk androgen deposition in free-living kittiwakes in two years (2003 and 2004) that varied in natural food availability. Chick survival was measured concurrently in other nests where eggs were not collected. In both years, supplemental feeding increased female investment in eggs by increasing egg mass. First-laid (“A”) eggs were heavier but contained less testosterone and androstenedione than second-laid (“B”) eggs across years and treatments. Yolk testosterone was higher in 2003 (the year with higher B chick survival) across treatments. The difference in yolk testosterone levels between eggs within a clutch varied among years and treatments such that it was relatively small when B chick experienced the lowest and the highest survival probabilities, and increased with intermediate B chick survival probabilities. The magnitude of testosterone asymmetry in a clutch may allow females to optimize fitness by either predisposing a brood for reduction or facilitating survival of younger chicks.

## Introduction

Animals benefit from adjusting reproductive effort to match environmental conditions [1]. Seabirds are not unusual in this regard – food availability during the breeding season is considered to be the primary determinant of reproductive success [2], [3]. In species with facultative brood reduction, one of the potential consequences of low food availability during the chick-rearing period is brood reduction via elimination of junior chicks. Brood reduction allows parents to optimize their reproductive success by balancing quantity and quality of offspring to match parental provisioning abilities [4], [5]. However, the vulnerability of seabird chicks to food availability during early development can be more subtle than outright mortality in the nest – while severe shortages result in death, even moderate shortages can compromise the quality and thereby survival of fledglings [6]–[8]. Females are not expected to support low quality or ‘marginal’ offspring if doing so compromises the fitness of chicks with higher reproductive value [5], [9]. Thus females should adjust brood reduction in response not just to food availability during chick-rearing, but to any factor that will contribute to their ability to provision and buffer chicks from fluctuations in food availability, including conditions experienced prior to chick-rearing. While the physiological mechanisms mediating maternal investment in eggs are not well understood, there is a large body of research indicating that the deposition of maternally derived androgens in egg yolk can facilitate adjustment of investment in offspring prior to chick-rearing [10].

Androgen deposition into egg yolk varies within and among individual female birds, and has both heritable and environmental components [11], [12]. The standard interpretation for patterns of yolk androgen deposition is that if androgens are higher in the first-laid eggs, they facilitate brood reduction via siblicide [13], while if androgens are higher in later-laid eggs within a clutch, they provide compensatory benefits to smaller, later-hatching chicks within the brood [10]. In species for which yolk androgen levels increase with laying order, exposure to yolk androgens alters chick phenotype, and the increased begging and growth that can be induced by high yolk androgens is usually proposed to help counter the disadvantage of hatching later and competing with older and larger siblings [14]–[17]. Although elevated yolk androgens may confer advantages to chicks such as enhanced ability to compete for food during the nestling and juvenile stage [18], [19] and do not appear to generate direct physiological costs in laying females [20], they are associated with costs to chicks. Reduced survival, growth and immunocompetence are tradeoffs that chicks from high-androgen eggs may face in some circumstances [10], [21]–[25]. However, maternal effects – including yolk androgen deposition – should benefit females, not chicks [26], [27]. Thus when females' interests conflict with chicks', females are likely to prevail and impose costly phenotypes on chicks if it benefits them [28]. Yolk androgens are nearly always discussed as either mitigating effects of hatching asynchrony or facilitating brood reduction, depending on the life history of the species. However, within a species that has facultative brood reduction, yolk androgen levels may be adjusted to promote fitness by either facilitating compensation for hatching asynchrony, or by promoting brood reduction [29], [30], depending on the context.

Black legged kittiwakes (*Rissa tridactyla*) are small, colonial, piscivorous gulls with facultative brood reduction. In the population we studied on Middleton Island in the Gulf of Alaska, AK, they frequently suffer food shortages during the reproductive season [31], [32]. They usually lay two-egg clutches that hatch asynchronously, although food shortages can increase the number of one-egg clutches or failed laying attempts [32]. In many nests, the B chick (from the second-laid egg) suffers a reduced growth rate and eventual death because it is outcompeted or directly killed by the A chick (from the first-laid egg) [33], [34]. Food clearly plays a role in brood reduction, because experimental food supplementation (where both parents and chicks are provided with ad libitum supplemental food for the duration of the chick-rearing period) permits a larger proportion of parents to successfully raise both chicks [35]. However, supplementally fed nests still show substantial interannual variability in junior (“B”) chick survival [34], [35], indicating that the immediate availability of food to chicks is not the sole factor contributing to brood reduction. While brood reduction may be affected by other factors specific to conditions during chick-rearing (i.e. weather conditions, predation pressure), it may also be affected by factors determined before chicks hatch. For example, the body condition of female birds at the start of the breeding season (a reflection of individual quality, prior experience and over-winter conditions) can interact with environmental conditions during breeding to affect investment in reproductive effort [36]. Deposition of androgens in yolk may be a mechanism by which females contribute to regulation of brood reduction in response to some combination of early food availability, their own condition, and pre-lay environmental conditions.

We conducted a two-year experiment with free-living kittiwakes to test whether access to supplemental food for approximately three weeks prior to egg-laying affected allocation of the steroid hormones testosterone (“T”) and androstenedione (“A4”) to A and B eggs of 2-egg clutches. We compared eggs from experimental birds to those from unfed controls in two years, and compared patterns of yolk androgen deposition to patterns of B chick survival to see whether yolk androgen levels were related to rates of brood reduction. The goal of the study was to compare the effects of pre-lay food supplementation on the intra-clutch dynamics of yolk androgen deposition and chick survival across years.

## Materials and Methods

### Study site and feeding

The study was conducted on free-living black-legged kittiwakes nesting on a modified radar tower on Middleton Island (Gulf of Alaska, 59°26′N, 146°20′W) in late May and early June of 2003 and 2004. All nest sites were on wooden ledges that were constructed on the outside face of the tower, and could be accessed from inside the tower. On the outside, nesting ledges were partitioned from one another with wood panels; kittiwakes chose and defended nest sites, built typical bowl-nests, laid eggs and reared young. Nest sites were individually fitted with a sliding one-way mirror that permitted observation and egg-collection at individual sites from within the tower. In addition, each site was equipped with a PVC feeding tube that allowed supplementary food provisioning to nest occupants (see Gill and Hatch, 2002 [32] for photographs and a more complete description of the study site and nesting ledge construction). Birds at this site were banded so that the identity of individual birds was known. Because all sites were partitioned, manipulations (feeding or egg collection) at one nest site did not disturb neighboring birds.

In both years, supplemental feeding started soon after birds' arrival to nest sites and continued until completion of the first clutch. Birds at “fed” nests were given fish 3 times per day at 0900, 1400 and 1800 for an average of 22 days prior to egg-laying (range: 11–35 days). At each feeding, whole thawed capelin (*Mallotus villosus*) were offered one at a time, until nest occupants refused to take additional fish. Number of fish consumed at each nest was recorded at each feeding. “Unfed” nests were visited and the feeding tubes manipulated on the same feeding schedule as fed birds, but birds were never allowed to consume supplemental fish. Capelin are the preferred prey for kittiwakes during both pre-lay and chick-rearing stages (chick and adult diets do not differ at this colony), and capelin availability is positively correlated with reproductive success in this population [31]. In both years, capelin for supplemental feeding were obtained from the same supplier, who targeted male capelin schools in Canada in late July, and provided adult fish of the same mass (average 25 g per fish). Despite supplemental feeding, adults continue to forage at sea in all years. The amount of supplemented food that fed birds choose to consume is negatively correlated with reproductive success in unfed birds (Hatch, unpublished data), and may serve as an indicator of natural food availability.

### Egg collection

One set of nests was used for egg collection. Eggs from pairs' first clutch of the season were collected. Nests were checked each morning and evening for eggs, which were immediately collected, weighed to the nearest gram, and replaced with foster eggs from nests at a different site that was not being used in the feeding study. Whole eggs were kept frozen (−4°C) and transported on dry ice to the University of Alaska, Fairbanks where they were kept at −20°C. In 2003, 2-egg clutches were collected from 10 pairs that were supplementally fed (hereafter, “fed”) and 10 pairs that were not supplementally fed (hereafter, “unfed”). We aimed to resample all nests the following year, however, not all focal birds were breeding in 2004. Therefore 3 out of 10 fed nests and 3 out of 7 unfed nests sampled in 2004 had not been sampled in 2003. The partial repeated sampling is addressed in the statistical methods.

### Yolk androgen assays

Eggs were thawed and yolk and albumen separated. Whole yolks were first weighed, and then homogenized using an electric tissue grinder in 15 ml of purified water. Aliquots were frozen until assay. For each sample, 20 mg of yolk-water homogenate was equilibrated with 2000 cpm of radiolabeled testosterone and androstenedione to assess percent hormone recovery after extraction. Steroids were extracted using the following version of the protocol used by Schwabl (1993) [37]: Samples were extracted twice with 4 ml diethyl ether, dried under continuous nitrogen gas flow in a water bath at 40°C, reconstituted in 1 ml 90% ethanol, and frozen for 24 h at −70°C. Androgens were separated using column chromatography. The ethanol was decanted and dried off using nitrogen. Samples were reconstituted in 0.5 ml 10% ethyl-acetate in iso-octane, and added to short columns of diatomaceous earth, with a water trap and glycol phase. Androstenedione was eluted in the first fraction, 2% ethyl-acetate in iso-octane, while testosterone was eluted in the third fraction, 20% ethyl-acetate in iso-octane. Average testosterone recovery was 59%, while average androstenedione recovery was 71%. Yolks were also assayed for corticosterone but levels were undetectable. Samples from 2003 and 2004 were each run in two separate radioimmunoassays (two assays per year). Samples from eggs in the same clutch were always run in the same assay. Treatments were randomly distributed across assays. For androstenedione and testosterone, intra-assay variability between duplicate samples was 3±0.2% and 2±0.2%, while inter-assay variation was 6±2% and 12±4%.

### Survival

A separate set of nests was used to monitor effects of supplemental feeding on chick survival (2003: unfed n = 49, fed n = 36; 2004: unfed n = 34, fed n = 46). These nests represented all nests from panels of the same radar tower where no manipulations other than feeding were being conducted. For these nests, food supplementation and control visitation (as described above) began at least two weeks before laying and continued until chicks fledged. Eggs were marked and chicks banded upon hatching (A and B chicks hatch at least one day apart [35]) such that A and B status of chicks was unambiguous. Adults and both chicks at fed nests were offered ad libitum fish at each feeding. Individual chicks were identified by metal leg bands. Presence of chicks was checked daily – if a chick was missing from the nest before 40 days post hatch, it was considered dead. If it was missing after that age it was considered to have fledged successfully. Second breeding attempts are rare and almost always fail - only first clutches were included in survival data.

### Statistical analyses

#### Egg mass and yolk androgens

To evaluate the relative contribution of inter-annual variation and the effect of supplemental feeding on investment in egg mass (g) and the yolk concentration of T and A4 (ng/g), we fitted linear mixed effects models with ‘egg’ (A vs. B egg), ‘treatment’ (fed vs. unfed) and ‘year’ (2003 vs. 2004) as fixed effects. To control for effect of lay date this parameter was included as a covariate in all models. To account for non-independence of eggs within a nest and the fact that most females were sampled in both years, models contained ‘nest’ within ‘female’ as a nested random term. Similarly, we fitted a linear mixed effects model to test for an effect of treatment and year on the within-clutch difference in T and A4 between B and A egg (T_diff_ and A4_diff_, respectively). In these models ‘female’ was included as a random effect to account for repeated measures of same individuals in both years.

In all cases, we used the information-theoretic approach to identify the most parsimonious model(s) of the set of candidate models [38], [39]. The set of candidate models comprised models with all possible combinations of explanatory variables and their two-way interactions. Models were ranked according to the Akaike Information Criterion adjusted for small sample size (AICc; [39]) the model with the lowest AIC_c_ was considered the best. We then calculated Akaike weights (*W_i_*) for each model using the formula: *W_i_* = (exp(−0.5* Δ_i_)/Σ^R^_r = 1_(exp(−0.5 * Δ_r_)). These values indicate the approximate probabilities that model *i* is the best model in the set of models considered, and the relative likelihood that model *i* is better than model *j* is *W_i_/W_j_* (evidence ratio). We determined the set that includes the best model in 95% of all samples (cumulative sum of *W_i_* = 0.95;[39] and used model averaging over this set of models to estimate effect sizes (β) and unconditional standard errors [39].

Model fits were assessed by examining diagnostic plots; hormone concentration data were log-transformed to meet condition on normality. All analyses were performed using R 2.10 [40].

#### Survival

In order to quantify the effect of the feeding treatment on the survival probability of A and B chicks in 2003 and 2004, we applied a generalized linear mixed effects model with a binary response (‘died’, ‘fledged’), a logit-link and a binomial error distribution. ‘Treatment’, ‘year’ and ‘chick’ (A vs. B) were included as fixed predictors in this model, ‘nest’ as the random factor to avoid pseudo-replication issues due to interdependence of siblings. Nests where fewer than 2 chicks hatched were not included in the analysis.

### Ethics statement

All research was conducted under state and federal permits (Alaska Department of Fish and Game Scientific Permit No. 03-002 and 04-061; U.S. Fish and Wildlife Service Scientific Collecting Permit No. MB789758-3, -5).

## Results

### Egg mass and supplemental food intake

**Figure 1:**
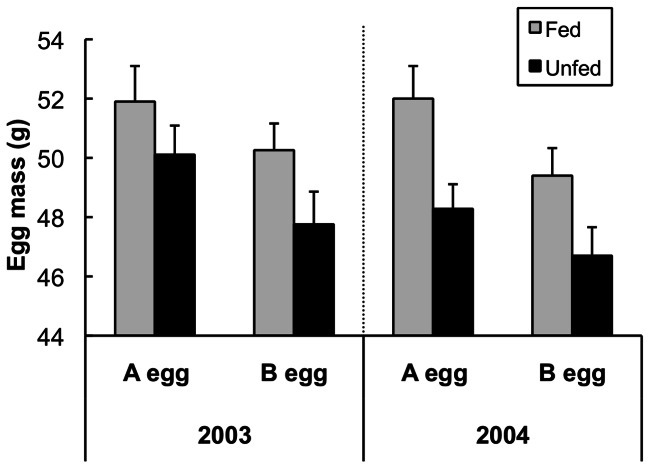
Effect of supplemental feeding on egg mass. Fresh mass of whole black-legged kittiwake eggs by laying order (A or B egg), treatment (fed or unfed) and year (2003 and 2004). N = 10 for all treatments and years except n = 7 for unfed 2004. Means ± SEM.

Model selection indicated a strong effect of laying sequence and feeding treatment on egg mass (Table 1). A eggs were heavier than B eggs (β = 2.04±0.33) regardless of treatment or year, and food supplementation caused an increase in egg mass in A and B eggs in both years (β = 2.52±1.23; Figure 1). Unfed eggs were heavier in 2003 compared to 2004 (β = 0.87±1.09; Figure 1). Heavier eggs in 2003 in unfed nests suggests that natural food availability at sea was higher in 2003, and is consistent with the fact that fed birds in 2003 chose to consume fewer supplemental fish than they did in 2004 (mean ± SE: 2003 = 110.6±18, 2004 = 167.5±17 fish/nest; t_1,18_ = −2.25, p = 0.04). In addition, the best model (and 3 out of the top 5 models) includes a ‘year’×‘treatment’ interaction suggesting a stronger effect of supplemental feeding on egg mass in 2004 than in 2003 (β = −0.67±1.82; Table 1).

**Table 1:** Top models explaining variation in egg mass, yolk androgens and survival, identified using the information-theoretic approach and Akaike's Information Criterion adjusted for small sample size (AICc).

Dependent	Model	k	ΔAIC_c_	W*_i_*	evidence ratio
					
**Egg Mass**					
	egg+treatment+year+(treatment×year)	8	0	0.179	1
	egg+treatment+year	7	0.609	0.132	1.4
	egg+treatment	6	1.140	0.101	1.8
	egg+treatment+year+(treatment×year)+(egg×year)	9	1.753	0.074	2.4
	egg+treatment+year+(treatment×year)+(egg×treatment)	9	1.808	0.072	2.5
**Yolk A4**					
	egg	5	0	0.672	1
	egg+treatment	6	2.714	0.173	3.89
	egg+year	6	4.321	0.077	8.67
	egg+lay date	6	6.458	0.027	25.26
	egg+treatment+year	7	7.27	0.018	37.9
**Yolk T**					
	egg+year	6	0	0.606	1
	egg+year+(egg×year)	7	3.587	0.101	6.01
	egg	5	3.640	0.098	6.17
	egg+year+treatment	7	4.437	0.066	9.2
	egg+year+treatment+(year×treatment)	8	4.837	0.054	11.23
**A4_diff_**					
	intercept only (null)	3	0	0.49	1
	treatment	4	2.095	0.172	2.85
	lay date	4	2.348	0.151	3.23
	year	4	2.495	0.141	3.48
	treatment+year	5	4.730	0.046	10.65
**T_diff_**					
	treatment+year+(treatment×year)	6	0	0.702	1
	treatment+year+lay date+(treatment×year)	7	3.847	0.103	6.84
	treatment	4	4.504	0.074	9.51
	treatment+year	5	4.869	0.062	11.41
	intercept only (null)	3	4.901	0.061	11.59
**Survival**					
	chick+year+treatment+(chick×year)	7	0	0.338	1
	chick+year+treatment	6	0.794	0.227	1.49
	chick+year+treatment+(chick×treatment)	7	1.618	0.151	2.25
	chick+year+treatment+(chick×treatment)+(chick×year)	8	1.653	0.148	2.29
	chick+year+treatment+(chick×treatment)+(year×treatment)	8	1.823	0.136	2.49

k is the number of parameters in the model, W_i_ is the Akaike weight calculated over all candidate models and the evidence ratio reflects the relative likelihood that model i is better than the best model (see Methods for details). “Egg” refers to A or B egg, “chick” refers to A or B chick.

### Yolk androgens

#### Androstenedione

**Figure 2:**
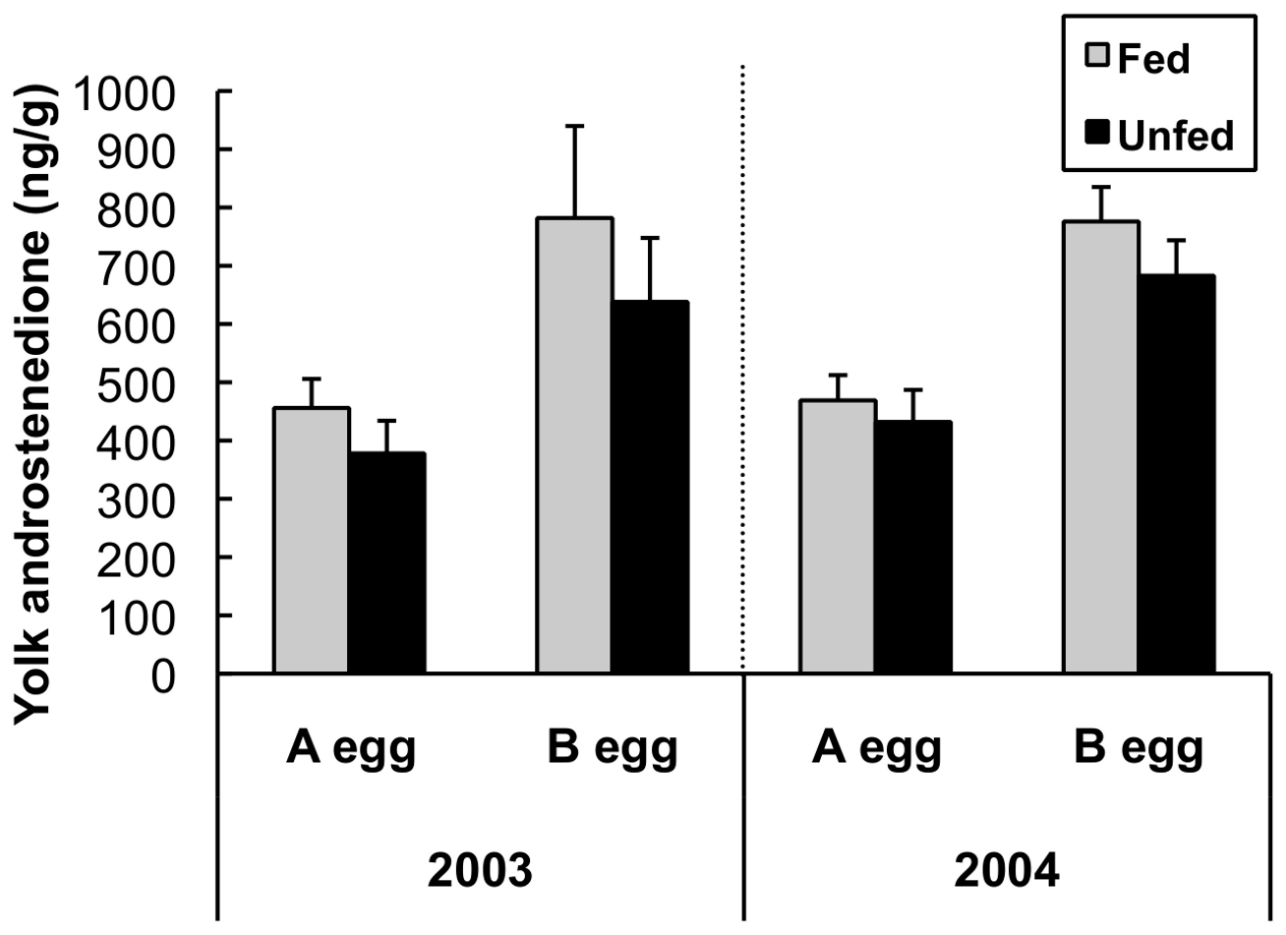
Effect of supplemental feeding on yolk androstenedione. Concentration of androstenedione in yolks of black-legged kittiwake eggs by laying order (A or B egg), treatment (fed or unfed) and year (2003 and 2004). N = 10 for all treatments and years except n = 7 for unfed 2004. Means ± SEM.

The model containing only laying order (“egg”) was the best supported for A4 levels (Table 1), with higher A4 concentration in B eggs than in A eggs (β = 0.49±0.06). Models including an additional effect of either year or treatment had limited support (Figure 2, Table 1). Likewise, there was no strong support for the difference in A4 between A and B eggs within a clutch (“A4_diff_”) to vary between years or with treatment; instead the null model (intercept only) was the model most supported by the information-theoretic analysis (Table 1).

#### Testosterone

**Figure 3:**
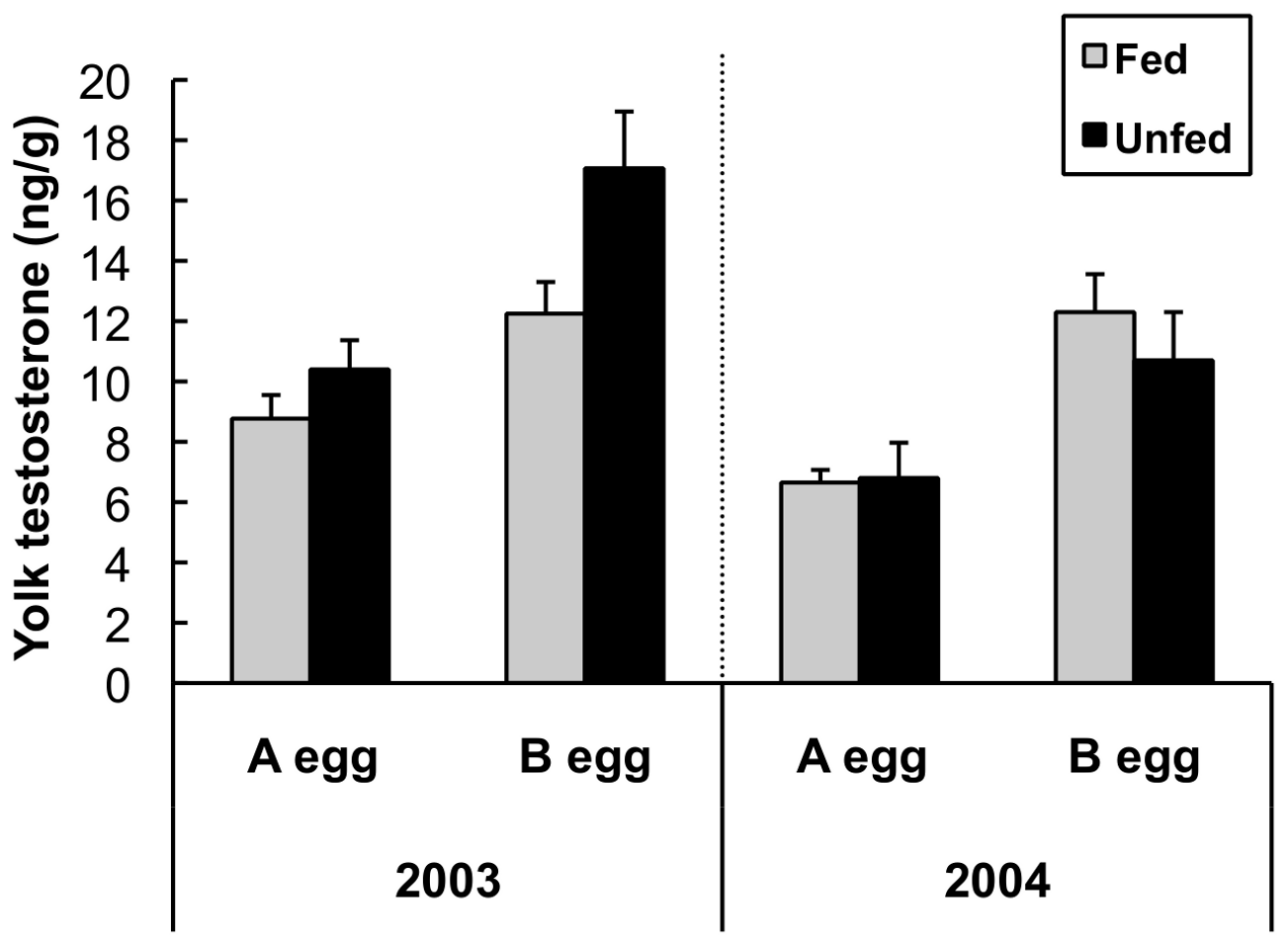
Effect of supplemental feeding on yolk testosterone. Concentration of testosterone in yolks of black-legged kittiwakes by laying order (A or B egg), treatment (fed or unfed) and year (2003 and 2004). N = 10 for all treatments and years except n = 7 for unfed 2004. Means ± SEM.

The concentration of testosterone depended on laying order (“egg”) and differed between years; the model containing these factors was the single best model, and four of the top five models also included both of these factors (Table 1). Parameter estimates indicated that B eggs contained more T than A eggs (β = 0.47±0.09), and testosterone was higher in 2003 than in 2004 (β = 0.26±0.12). However, there was no apparent effect of treatment on testosterone levels (β = 0.02±0.04); models including treatment had only limited support (ΔAICc>9, Table 1, Figure 3).

**Figure 4:**
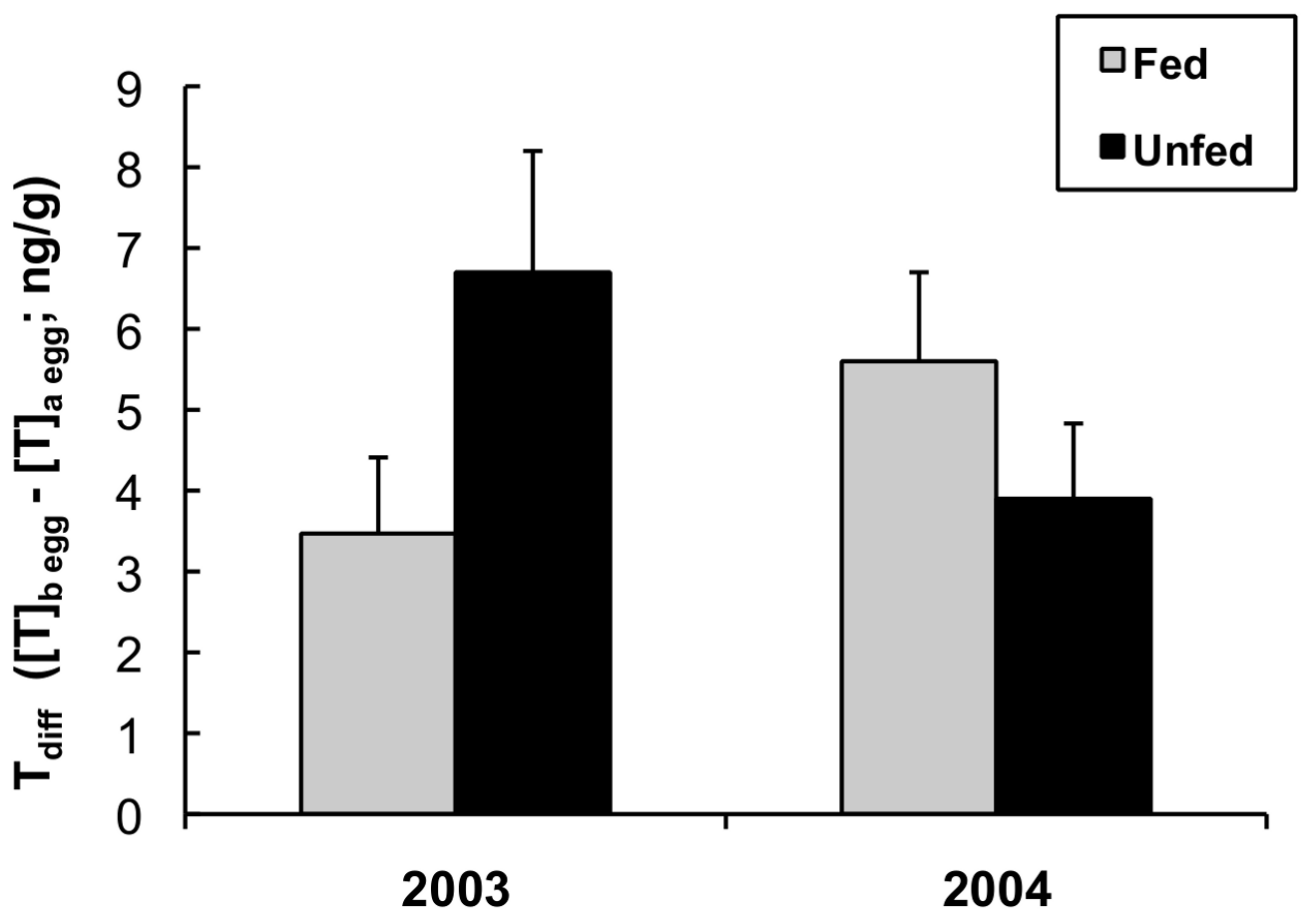
Effect of supplemental feeding on differences in yolk testosterone within a clutch. The difference in yolk testosterone levels between B and A eggs (“T_diff_” = B egg T (ng/g)−A egg T (ng/g)) in fed and unfed black-legged kittiwake nests in two years). N = 10 for all treatments and years except n = 7 for unfed 2004. Means ± SEM.

Variation in the difference in T between A and B eggs within a clutch (“T_diff_”) was best explained by treatment, year, and an interaction of treatment and year; this interaction appeared in the top two models, and models that did not include this interaction were >9 times less well supported (Table 1). While in 2003 T_diff_ was higher in unfed than in fed nests (β = 2.99±2.06), in 2004 it was lower in unfed compared to fed nests (β = −1.68±2.44; Figure 4; Table 1).

#### Chick survival

**Figure 5:**
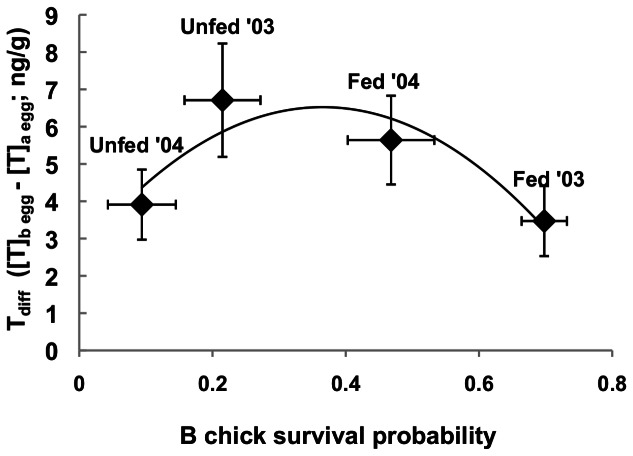
The relationship between differences in yolk testosterone within a clutch (“T_diff_”) and B-chick survival. T_diff_ (the difference in yolk testosterone between B and A eggs) is related non-linearly to the probability of survival for B chicks in each year/treatment. Yolk testosterone and survival data were collected from different nests. For T_diff_, n = 10 for all treatments and years except n = 7 for unfed 2004. For B chick survival in 2003, unfed n = 49, fed n = 36; for 2004, unfed n = 34, fed n = 46. Mean ± SEM.

In 2003 and 2004 model selection indicated strong effects of chick rank within a brood, treatment and year on survival (5 of 5 top models; Table 1). Survival of B chicks was always lower than that of A chicks (β = −1.81+/−0.49) and was higher in fed nests than in unfed nests in both years (β = 2.00±0.42; Figure 5). Within treatment, B chick survival was higher in 2003 than in was in 2004 (β = 1.14±0.49; Figure 5).

## Discussion

In black-legged kittiwakes, a complex suite of factors determines yolk androgen levels. Pre-lay food supplementation allowed females to increase investment in reproduction – A and B eggs were heavier in supplemented nests in both years (Figure 1). Supplemental feeding also affected the magnitude of the difference in testosterone between A and B eggs within a clutch (“T_diff_”; Figure 4), but did so in opposite ways in two years with contrasting patterns in natural food availability. Feeding decreased T_diff_ relative to controls in 2003 (higher natural food availability), but increased it in 2004 (lower natural food availability).

2003 offered higher natural food availability (at sea) than 2004 for kittiwakes breeding on Middleton Island, as reflected in both fed and unfed birds. In 2003, fed birds relied less on supplemental food, choosing to obtain more food at sea (S. Hatch, unpublished), while unfed birds in 2003 laid heavier eggs (Figure 1), successfully fledged more B chicks compared to unfed birds in 2004 (Figure 5), and had higher overall reproductive success [31].

### Yolk androgens

Levels of yolk androstenedione (“A4”) in eggs of the first clutch appear to be relatively fixed: in both years the main source of variation was the difference between A and B eggs (Figure 2). Thus in black-legged kittiwakes, A4 levels appear to be less sensitive to environmental variation than testosterone. Another study that measured yolk A4 (but not testosterone) from fed and unfed birds on Middleton I. in a single year (2002) confirmed our finding that feeding had no effect on androstenedione levels in the first clutch, but in replacement clutches, a) androstenedione was higher in the replacement clutch and b) feeding significantly reduced yolk A4 levels in the B eggs of replacement clutch [41]. Variation in yolk A4 is challenging to interpret because the effects of A4 in yolk on chick phenotype remain mostly uninvestigated (most studies that have manipulated yolk A4 concurrently manipulated yolk T). A4 may exert direct effects on phenotype, may serve as a precursor for both T and estradiol, or may be inactivated by embryos [42]. Because little is currently known about the mechanisms of action or consequences of A4 in egg yolk, no obvious explanation exists for why there is so much more A4 than T present in yolk, or why B eggs consistently contained more A4 than A eggs; however, the same pattern has been found in multiple species [12], [43], [44]. This pattern may be the result of constraints associated with the physiology of egg-laying [45], [46], and not a reflection of selection pressure. Regardless, A4 deposition into eggs of the first clutch appears to be insensitive to both interannual differences and experimental manipulation of food availability in kittiwakes (Figure 2).

In contrast to yolk A4, yolk T allocation varied both between eggs and years – it was higher in B eggs, and higher in 2003 (the year with better natural foraging conditions; Figure 3). Although elevated T in a better year is consistent with the hypothesis that females should only invest in active, competitive chicks when they can afford to meet the energetic demands of the phenotype, this result appears to be in contrast with a supplemental feeding study in lesser black-backed gulls (*Larus fuscus*). Verboven et al. (2003) found that females gulls fed with chicken eggs during pre-lay appeared to deposit less androgen in their eggs, rather than more [43]. However, their results may not be comparable to those presented here, since yolk steroid levels in the *Larus* study were determined from eggs that had been incubated for 4 days [43], at which point substantial metabolism of yolk steroids has already occurred [47].

While pre-lay food availability clearly affected at least one measure of reproductive investment (egg mass), it is not the primary factor determining yolk androgen concentrations in kittiwakes. Interannual variation in egg contents may be a function of conditions experienced by birds prior to breeding (i.e. over winter food availability) or may be driven by aspects of pre-lay conditions other than food availability. Interestingly, the only other multi-year study of yolk androgen deposition in seabirds found no interannual differences in T or A4 in two auklet species with single-egg clutches, despite evidence for large interannual differences in food availability and reproductive success [48]. This suggests that variation in yolk androgen levels generated in response to environmental conditions may be primarily relevant to intra-clutch dynamics.

### Intra clutch differences in androgens

The magnitude of intra-clutch differences in yolk androgens may be more relevant than absolute levels. As previously found in black-legged kittiwakes [41] yolk testosterone was consistently higher in B eggs in the present study (Figure 3). Although androgens are higher in eggs later in the laying sequence in many species (reviewed in [10]), the magnitude of this difference is a neglected but potentially important measure. Differences in T between B eggs and A eggs are likely to have consequences for intra-brood competition and fitness. Several studies have shown that size asymmetry is a critical component in the intra-brood dynamics of aggression and food monopolization. The more closely matched nest mates are in size, the more intense the competition and the more likely the junior chicks are to obtain food and increase survival probabilities [49], [50]. However, aggressive behavior can compensate for age and size discrepancies between nest mates [51]. Elevated yolk androgens in B eggs increases competition between siblings by increasing aggression and dominance [15], [18], [19], behavioral alterations that functionally reduce the size asymmetry between A and B chicks. Thus we propose that the greater the magnitude of the androgen difference between eggs, the more intense the competition is likely to be and the better the B chick can compensate for its smaller size and lower rank within the nest.

Large intra-clutch differences in yolk testosterone, and therefore increased competition, are only likely to be beneficial under certain conditions. In particular, experiments in free-living black-headed gulls (*Larus ridibundus*) (which lay 3-egg clutches but have patterns and levels of yolk androgen deposition similar to kittiwakes) demonstrated that if differences in androgen levels between A eggs and B/C eggs were large, the growth of the junior chicks was enhanced at the expense of the A chick. In contrast, in nests where differences in androgen levels among eggs were eliminated, A chicks grew faster at the expense of their younger siblings [15]. Although food availability was not measured or manipulated in that study, the results suggest that the relative benefits of establishing a large or small androgen difference between eggs in a clutch should be affected by parental provisioning ability.

While females are likely able to adjust brood reduction via provisioning decisions during chick-rearing, the high variability between years in chick survival in kittiwake nests where both parents and chicks are supplementally fed (and therefore not food limited) indicates that females may be adjusting their broods' predisposition for reduction prior to chick-rearing. We propose that the magnitude of T_diff_ may contribute to this predisposition. If there is plenty of food (e.g. for chicks from fed nests in 2003, the good year; Figure 5), kittiwake A chicks are well-fed and less aggressive, and the B chick is likely to perform well regardless of its competitive predisposition [34]. In fact, in years with very high natural food availability, A and B chicks from fed nests on Middleton I. can have similar survival and growth rates [35]. Under such circumstances females are unlikely to benefit from increasing levels of competition by generating a large intra-clutch difference in yolk androgens, and potentially incurring the reproductive costs of increased inter-sibling aggression – T_diff_ is low (Figure 5).

In contrast, when provisioning ability is low (unfed birds in a poor year (2004); Figure 5), brood reduction is likely to occur rapidly. For example, in previous low-food years at Middleton Island, 50% of B chicks from unfed nests died in the first 10 days post-hatch, and less than 30% survived until fledging [35]. The reproductive value of B chicks that do fledge is much lower than that of A chicks [52] – specifically Cam et al. (2003) demonstrated that the proportion of kittiwake B fledglings that survive to recruit to the breeding population is dramatically lower than that of A chicks [53]. Given the low probability that the B chick will contribute to her fitness, a female should benefit from reducing the competitive intensity between A and B chicks when her provisioning ability is low [52] –T_diff_ should be low (Figure 5). Reducing the difference in T levels between eggs may serve to minimize the degree to which a doomed B chick can compromise the quality of the A chick by forcing it to compete aggressively for food [34]. In this scenario, the B chick would serve as reproductive insurance should the A chick not survive [9].

We propose that it is primarily when provisioning ability is intermediate or unpredictable that females may benefit from maximizing competition between chicks, because the consequences of enhanced competition will be dependent on the conditions that emerge during chick-rearing. A large difference between eggs in yolk T when conditions are uncertain may be a bet-hedging strategy, mediated by intra-brood competition, that helps females ‘win’ any parent-offspring conflict that might arise should food availability become insufficient [54]. Under this scenario, similar to that proposed by Royle et al. [23], B chicks will be more competitive and be more likely to survive if conditions improve. If conditions remain poor or decline further, however, the increased aggression [15] is likely to impose costs [34], potentially hastening brood reduction and permitting the A chick to monopolize food resources sooner.

The degree to which laying kittiwakes can use early season environmental cues to anticipate food availability during chick-rearing is ambiguous [55]. However, the results of this study lend support to the hypothesis that female physiology during egg-laying contributes to the probability of brood reduction during chick-rearing [29]. The year×treatment interaction for T_diff_ indicates that food supplementation alone did not dictate patterns of yolk androgen deposition (Table 1), but interacted with other environmental factors. The highest T_diff_ was established under conditions that ultimately yielded intermediate B chick survival probabilities (Figure 5). The highest and lowest survival probabilities observed were associated with the lowest T_diff_ (Figure 5). Although there is evidence that the effects of yolk T on phenotype increase with dose [56], they may not be linear across the entire physiological range. However, further studies that experimentally manipulate T_diff_ and/or generate mismatches between pre-laying conditions and food availability during chick-rearing would provide insight into the role of pre-lay maternal influence on brood reduction.

## Conclusions

Our data demonstrate that patterns of yolk androgen deposition by kittiwakes can be affected by food availability. However food availability alone does not fully explain variability in this maternal effect. Yolk androgen deposition is also likely to be affected by longer-term aspects of female condition or quality, since experiments manipulating food availability to birds during egg-laying have yielded complex and inconsistent effects on androgen deposition [41], [43], [57]. Previous studies have suggested that patterns of yolk androgen allocation are a species-specific trait which serves either to mitigate hatching asynchrony or to enhance brood reduction, based on the life-history of species under consideration [13], [43], [58]–[60]. We propose that yolk androgen allocation has the potential to serve either function within a species, particularly those with facultative brood reduction, and that consideration of the magnitude of the differences in yolk androgens among eggs within a clutch will prove relevant to chick behavior and maternal fitness.
